# The Effect of Magnetic Fields on Wound Healing: Experimental Study and Review of the Literature

**Published:** 2008-07-25

**Authors:** Steven L Henry, Matthew J Concannon, Gloria J Yee

**Affiliations:** Division of Plastic Surgery, University of Missouri Hospital & Clinics, Columbia, MO

## Abstract

**Objective:** Magnets are purported to aid wound healing despite a paucity of scientific evidence. The *purpose* of this study was to evaluate the effect of static magnetic fields on cutaneous wound healing in an animal model. The literature was reviewed to explore the historical and scientific basis of magnet therapy and to define its current role in the evidence-based practice of plastic surgery. **Methods:** Standardized wounds were created on the backs of 33 Sprague-Dawley rats, which were divided into 3 groups with either a 23 gauss magnet (group 1), a sham magnet (group 2), or nothing (group 3) positioned over the wound. The rate of wound closure by secondary intention was compared between the groups. Literature review was conducted through searches of PubMed and Ovid databases for articles pertinent to magnets and wound healing. **Results:** Wounds in the magnet group healed in an average of 15.3 days, significantly faster than those in either the sham group (20.9 days, *P* = .006) or control group (20.3 days, *P* < .0001). There was no statistically significant difference between the sham and control groups (*P* = .45). **Conclusions:** An externally applied, low-power, static magnetic field increases the rate of secondary healing. Review of the literature reveals conflicting evidence regarding the use of magnetic energy to aid the healing of bone, tendon, and skin. Level I studies are lacking and difficult to execute but are needed to define conclusively the role of magnets in clinical practice.

Throughout history physicians have sought techniques to facilitate wound healing. From salves and potions to hyperbaric oxygen chambers, the means by which physicians have attempted to manipulate the wound healing process have been innumerable and, despite the claims of their proponents, oftentimes ineffectual.^1^,^2^

One popular yet controversial modality is magnet therapy. Particularly in alternative medicine circles, magnets have been touted to promote the wound healing process with claims of decreased pain, accelerated healing time, and increased scar strength. However, these claims have little support in the scientific literature^3^,^4^ and the use of magnetic field energy for medical treatment remains limited.

In this study we sought to investigate scientifically the effect of an externally applied, low-power, static magnetic field on the rate of wound healing in a rat model. We also reviewed the literature to explore the historical and scientific basis of magnet therapy and to define its current role in evidence-based medicine as it pertains to plastic surgeons.

## METHODS

**Figure 1:**
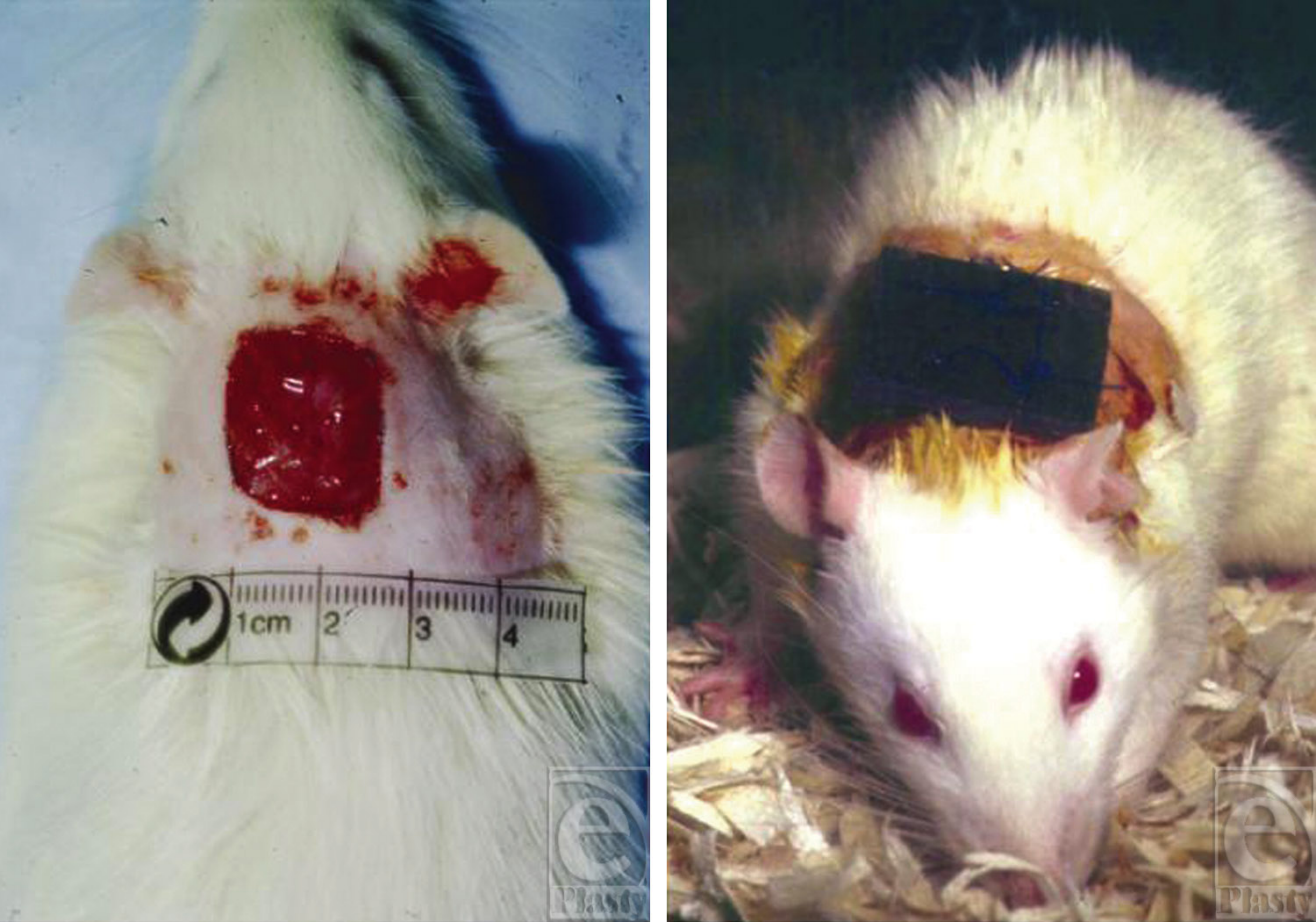
A 23 gauss magnet measuring 2 × 2 cm was placed over the wound on the back of Sprague-Dawley rats, directly on top of the occlusive dressing.

Standardized wounds were created on the backs of 33 Sprague-Dawley rats. These wounds measured 1.5 × 1.5 cm and were produced under sterile conditions by excising skin, subcutaneous tissue, and panniculus carnosus.After achieving hemostasis, the wounds were covered with an occlusive dressing. The animals were then equally divided into 3 groups. In *group 1*, a 23 gauss magnet measuring 2 × 2 cm was placed over the wound directly on top of the occlusive dressing (Fig 1) (This magnetic strength was chosen to be commensurate with commercially available products marketed for “medical” use). In *group 2*, a piece of leather of the same dimensions was likewise placed over the wound to serve as a sham magnet. *In group 3*, nothing was placed on the wound (other than the occlusive dressing). The wounds were allowed to heal by secondary intention and the time to complete closure was recorded for each animal. The *t* test was used to compare the mean healing rates of each group.

In the review of the literature, searches of PubMed and Ovid databases were performed. Articles pertaining to magnets and wound healing particularly with regard to bone, skin, and tendon were perused.

## RESULTS

**Figure 2:**
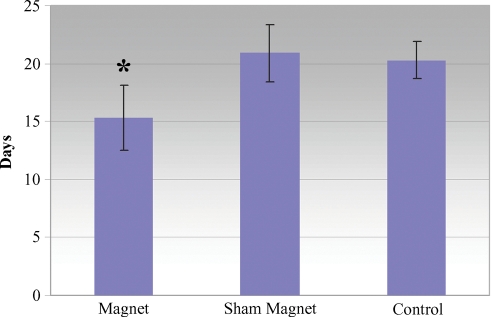
Graph comparing the mean time towound closure in the group treated with magnets to those treated with sham magnets or nothing.

The mean time to wound closure in the group treated with magnets was 15.3 ± 2.8 days compared with 20.9 ± 2.5 days for the sham magnet group and 20.3 ± 1.6 days for the control group (Fig 2). This represents a 27% reduction in healing time relative to the sham group and a 25% reduction relative to the control group. Both comparisons were highly statistically significant (*P* = .006 vs sham group and *P* < .0001 vs control group). There was no statistically significant difference between the sham and control groups (*P* = .45).

## DISCUSSION

The results of this study suggest that exposure to a static magnetic field increases the rate of cutaneous wound healing by secondary intention and provide further testimony to the notion that magnetic fields can influence the physiology of the human body. However, as the following discussion reveals, the precise mechanism and clinical applicability of this effect are still poorly defined.

The earliest reported use of magnetic therapy to aid wound healing dates to the 1600s, when electrically charged gold leaf was applied to smallpox lesions in an attempt to prevent scarring.^1^ Throughout the following centuries magnetic energy was propounded as a treatment for innumerable ailments and conditions, usually without substantiation of any kind. Today, however, at least 1 application, the promotion of bone healing has garnered strong scientific support and widespread clinical acceptance. The genesis of this application began in the 1950s, when Fukuda and Yasuda in Japan described the piezoelectric effect of bone, in which an electrical potential is produced as a response to mechanical stress.^5^ Subsequent investigations elucidated the numerous actions of electromagnetic energy on bone including effects on cellular calcium and calcification,^6^,^7^ collagen and proteoglycans,^8^,^9^ and angiogenesis.^10^ Clinical investigations proved the benefit of electromagnetic therapy in the treatment of delayed unions,^11^–^14^ difficult fractures,^15^ and osteotomies.^16^,^17^ The electrical current and electromagnetic field produced by a bone stimulator is a common application of this concept.

Although there is ample experimental and clinical evidence supporting the use of magnetic fields to aid bone healing, its application for soft tissue healing, including skin and tendons, is still ambiguous. Promising research along these lines was first produced in the 1960s by Becker. Studying amphibians, he described the presence of an electromagnetic skin circuit, alterations which accompanied limb regeneration.^18^ Borgens et al confirmed that this current is essential for amphibian limb regeneration and that its reversal induces limb degeneration.^19^,^20^ In a study involving limb amputations in frogs, a species that does not naturally produce this current and that is normally incapable of limb regeneration, induction of this current stimulated the regeneration of a rudimentary limb that included cartilage, nerve, and skin tissues.^20^ These skin circuits have been identified in humans and are similar in magnitude to those demonstrated in amphibians.^21^ Given this fact, it is plausible that external magnetic therapy could influence soft tissue healing in humans as well.

Several laboratory studies support this theory and most implicate a vascular mechanism of action. For example, Tepper et al applied pulsed electromagnetic energy to endothelial cell cultures and demonstrated a marked increase in proliferation and tubulization. They also reported a substantial increase in the expression of fibroblast growth factor 2 (FGF-2), a potent stimulator of angiogenesis, and showed that anti-FGF-2 antibodies inhibited the effects of the electromagnetic energy.^22^ This upregulation of FGF-2 in endothelial cells exposed to pulsed electromagnetic fields was recently confirmed by Callaghan et al.^23^ Roland et al used pulsed magnetic energy to stimulate neovascularization in a rat model.^24^ Weber et al demonstrated increased survival of rat groin composite flaps supported by an arterial loop, again showing that pulsed magnetic fields promote neovascularization.^25^

Less consistent results have been reported in investigations of the direct effect of magnetic energy on cutaneous blood flow. Miura and Okada showed that the arterioles of frogs' webs dilate in response to pulsed electromagnetic radiation. This effect was shown to be independent of heat and was postulated to involve the modulation of calcium balance in vascular smooth muscle cells.^26^ Gmitrov et al observed increased blood flow when a static magnetic field of 2500 gauss was applied to rabbit ears,^27^ whereas Smith et al noted significant arteriolar vasodilatation when pulsed electromagnetic energy was applied to the cremaster muscle of rats.^28^ However, in a series of studies Ichioka et al demonstrated decreased cutaneous blood flow and temperature in rats exposed to an 8 tesla (80,000 gauss) superconducting magnet,^29^–^31^ whereas Mayrovitz and Groseclose found that a 4000 gauss static magnet reduced perfusion in the fingers of human volunteers.^32^

Several investigators have employed a rat model similar to ours to examine the effect of magnetic fields on cutaneous wound healing, yet have produced conflicting results. Leaper et al studied the effect of 400 gauss magnetic foil (a static field) applied over wounds. They found no influence on wound healing rate, collagen content, or tensile strength.^33^ Patino et al demonstrated faster healing in wounds treated intermittently with pulsed electromagnetic fields of 200 gauss.^34^ Similar benefits were found by Callaghan et al in diabetic mice.^23^ Strauch et al observed accelerated healing and higher tensile strength in rat wounds exposed to pulsed electromagnetic fields.^35^ On the other hand, Milgram et al found that pulsed magnetic energy did not have a significantly beneficial effect on the rate of wound healing in a rat model.^36^

The data regarding magnet therapy for tendon healing are even more ambiguous. Greenbough applied pulsed electromagnetic fields to repaired flexor tendons in rabbits and found no benefit in terms of tensile strength or adhesion formation,^37^ whereas Robotti et al showed that pulsed electromagnetic fields decrease tensile strength and increase adhesions after tendon repair in chickens.^38^ These studies are in stark contrast to that of Strauch et al who recently demonstrated a 69% increase in tensile strength in repaired Achilles tendons in rats. They emphasized the importance of using a pulsed magnetic field of low amplitude (0.1 gauss) designed to maximize the effect on calcium ions, which, in theory, enhances the calcium-dependent activation of growth factors.^39^

Interestingly, our protocol employed a static magnetic field (23 gauss) that wasrelatively weak compared with those used in many of the aforementioned studies, yet our results indicate a relatively profound effect. Other examples of seemingly contradictory results abound in the literature, many of them presented in this discussion. Most modern investigators believe that pulsed magnetic energy is more effective than static but as seen above both successes and failures have been observed with both modalities. From a practical perspective, the ease of use and affordability of a small static magnet is appealing compared with a relatively cumbersome and expensive pulsed magnetic field generator.

Review of the magnet literature is frustrating not only for the contradictory results of the in vitro and animal studies but also for the lack of well-designed, well-executed clinical trials in humans. Unfortunately, a truly randomized trial, with perfectly matched cohorts, is almost impossible to achieve in the setting of wounds, particularly those involving bone, tendon, and/or skin. Level I evidence regarding the use of magnets, at least as it pertains to plastic surgery, is therefore likely to remain elusive.

## CONCLUSION

The application of a low-power, static magnetic field over an excisional wound appears to increase the rate of healing by secondary intention. Review of the literature reveals substantial evidence demonstrating a beneficial effect of magnetic therapy on bone healing but mixed results on tendon and skin healing. Recent laboratory and animal studies point to a vascular, and possibly a calcium-based, mechanism of action. Level I studies are lacking and difficult to execute but are necessary to define conclusively the role of magnets in clinical practice.
